# Time Course of Motor Activity Wake Inertia Dissipation According to Age

**DOI:** 10.3390/clockssleep4030032

**Published:** 2022-08-30

**Authors:** Lorenzo Tonetti, Miranda Occhionero, Marco Fabbri, Sara Giovagnoli, Martina Grimaldi, Monica Martoni, Vincenzo Natale

**Affiliations:** 1 Department of Psychology “Renzo Canestrari”, University of Bologna, 40127 Bologna, Italy; 2 Department of Psychology, University of Campania Luigi Vanvitelli, 81100 Caserta, Italy; 3 Department of Experimental, Diagnostic and Specialty Medicine, University of Bologna, 40138 Bologna, Italy

**Keywords:** wake inertia, motor activity, actigraphy, age

## Abstract

The time course of motor activity sleep inertia (maSI) dissipation was recently investigated through actigraphy in an everyday life condition from middle childhood to late adulthood. Motor activity sleep inertia was dissipated in 70 min, and the sleep inertia phenomenon was more evident in younger participants than in older participants. The aim of the current secondary analysis of previously published data was to examine, within the same sample, the time course of motor activity wake inertia (maWI) dissipation, i.e., the motor pattern in the transition phase from wakefulness to sleep, according to age. To this end, an overall sample of 374 participants (215 females), ranging in age between 9 and 70 years old, was examined. Each participant was asked to wear an actigraph around their non-dominant wrist for one week. The variation in the motor activity pattern of the wake–sleep transition according to age was examined through functional linear modeling (FLM). FLM showed that motor activity wake inertia dissipated around 20 min after bedtime. Moreover, a lower age was significantly associated with greater motor activity within the last two hours of wakefulness and the first twenty minutes after bedtime. Overall, this pattern of results seems to suggest that maWI dissipation is comparable to that of maSI.

## 1. Introduction

From a chronobiological point of view, sleep behavior should be considered within the circadian cycle of alternating wakefulness and sleep. In this framework, the main reference model is the two-process model [1]. This model provides a process, named S (Sleep), which refers to the need for sleep after a long period of wakefulness, and it is inspired by homeostatic principles. The second process, named C (Circadian), drives several functions, such as body temperature, within a period that is close to 24 h. The interaction between these two processes modulates alertness levels and sleep behavior [2]. Overall, the two-process model works quite well. For example, it has excellent predictive abilities in case of sleep deprivation. However, it does present some critical aspects when the transition phases (sleep–wake and wake–sleep) are observed; these are moments characterized by a considerable degree of functional uncertainty in terms of different regulatory systems.

According to the two-process model, upon awakening in the morning, the organism, after having dissipated its need for sleep, should already be at its best. However, it has been found that during wakefulness immediately following daytime sleep, people are not yet performing at their best [3,4,5]. This phenomenon is named sleep inertia. The transition phase presents electrophysiological changes that occur more quickly but, from a cognitive and behavioral point of view, functional recovery is rather slow. The sleep–wake transition has a well-known research tradition. During sleep inertia, alertness levels are low, and cognitive performance, such as that relating to memory, reasoning, and visual search, is impaired. Typically, sleep inertia takes between 15 and 30 min to dissipate, with performance improving over time [6,7,8]. However, based on the type of variable being considered, some studies have found that, after awakening, it may take as long as three hours for the sleep inertia phenomenon to plateau or stabilize, even after a nominally sufficient sleep opportunity (e.g., 8 h nocturnal sleep) [9].

The transition phase from wakefulness to sleep is referred to as falling asleep and is evaluated using specific electrophysiological changes which allow us to evaluate sleep-onset latency [10,11,12]. Before, and while, these variations are triggered, a series of behavioral shifts, revealing a decrease in responsiveness to external sensory stimuli, are also observed until a complete layoff of environmental response occurs. In analogy to how the previous transition phase is identified, we could call this equally important transition phase wake inertia. While sleep onset has received increasing attention in the sleep research field, pre-sleep behavioral studies are often limited to clinical populations, such as those with insomnia, and refer to those practices that are categorized as sleep hygiene [13,14,15,16]. It is commonly experience that the pre-sleep period is characterized by a series of motivational drives that can significantly modulate sleep [17,18]. In several animal species, the engagement of pre-sleep behaviors has also been documented in terms of changes in neuronal function and interpreted as an important evolutionary adaptation [19]. In humans, pre-sleep behaviors are thought to facilitate the transition from wakefulness to sleep by reducing alertness and limiting exposure to environmental, activating stimuli. However, from a psychophysiological point of view, the behavioral changes that precede sleep onset still remain a poorly studied phenomenon.

Recently, a particular aspect of SI, concerning the time course of motor behavior, has been investigated: motor activity sleep inertia (maSI) [20]. Motor behavior can be assessed through actigraphy, a method that allows researchers to collect numerous data in an ecological context. In this research, motor activity sleep inertia was dissipated in just over an hour, and the motor activity sleep inertia phenomenon was much more evident in younger rather than in older participants. We believed that the wake inertia phase could also be described using actigraphy, a method that allows one to examine the participant at home for several consecutive days. To our knowledge, there are no data in the literature on motor behavior in the transition period preceding falling asleep, which we could define as motor activity wake inertia (maWI), a pre-sleep period that precedes, and should favor, falling asleep.

For this reason, we decided to assess pre-sleep motor behaviors in the wake-to-sleep transition. We carried out a secondary analysis of previously published data by reanalyzing the sample investigated in the previous maSI study [20], i.e., a large sample of normal sleepers, ranging from children to older people. There were two aims: (1) to evaluate the time course of maWI dissipation; (2) to evaluate a possible age effect. Based on our knowledge of the actigraphic cut-off value for sleep-onset latency, which should be within 14 min [21], we expected maWI to be dissipated within 15 min. Regarding the second aim, we expected age to modulate the phenomenon of maWI. In particular, based on previous results on maSI, we made a second hypothesis that the maWI phenomenon would be more evident in young people, as the pre-sleep behavior of this age group is characterized by a higher activation level than that of the adult population.

## 2. Materials and Methods

### 2.1. Participants

An overall sample of 374 healthy participants (215 females and 159 males), ranging in age between 9 and 70 years old, was examined. The mean, standard deviation, median, inferior quartile and superior quartile of age were 22.21, 15.41, 17, 13 and 23 years, respectively. The participants did not use drugs with effects on sleep and/or cognition and kept regular sleeping habits. This dataset was examined in previous publications [20,22] and was assembled using data gathered from previous studies [21,23,24,25,26,27].

### 2.2. Actigraphy

The actigraph model Actiwatch AW64 (Cambridge Neurotechnology Ltd., Fenstanton, UK) was used to monitor the sleep/wake cycle. The hardware of the actigraph was composed of a piezoelectric accelerometer with >0.05 g sensitivity. The frequency of sampling was at 32 Hz with filters at 3–11 Hz. The Actiwatch Activity and Sleep Analysis software (version 5.32; Cambridge Neurotechnology Ltd., Fenstanton, UK) was used to initialize the actigraphs to collect motor activity data in 1 min epochs and to score the record using the medium threshold sensitivity setting [21,28]. The same software was also used to extract the motor activity counts, minute by minute.

### 2.3. Motor Activity Pattern of Wake–Sleep Transition 

Using the data regarding bedtime and the minute-by-minute motor activity obtained with the software Actiwatch Activity and Sleep Analysis (version 5.32; Cambridge Neurotechnology Ltd., Fenstanton, UK), we explored the raw motor activity pattern of the wake–sleep transition for each recorded day, extracting the motor activity counts from 120 min before bedtime up to 180 min after bedtime on school/work days only.

### 2.4. Procedure

Participants were required to wear the actigraph around the wrist of their non-dominant hand for at least seven consecutive days. They were included in the current secondary analysis of previously published data only if the actigraph was used for at least four school/work days with no missing data in the 120 min before bedtime and an overall number of missing 1 min epochs per day of lower than 14.

Moreover, participants were instructed to push the event marker button on the actigraph to signal bedtime (lights off) and get-up time (lights on).

### 2.5. Statistical Analyses

Functional linear modeling (FLM) [29] was used to examine the variation in the motor activity pattern of the wake–sleep transition, according to age, within the time interval starting from 120 min before bedtime up to 180 min after bedtime.

First, the raw motor activity pattern was transformed into a functional form by using the Fourier expansion model. Second, the non-parametric permutation F-test was applied to determine if and when the motor activity pattern of the wake–sleep transition significantly changed, according to age, within the previously described time interval.

FLM was performed using the “Actigraphy” package in R statistical software.

## 3. Results

**Figure 1:**
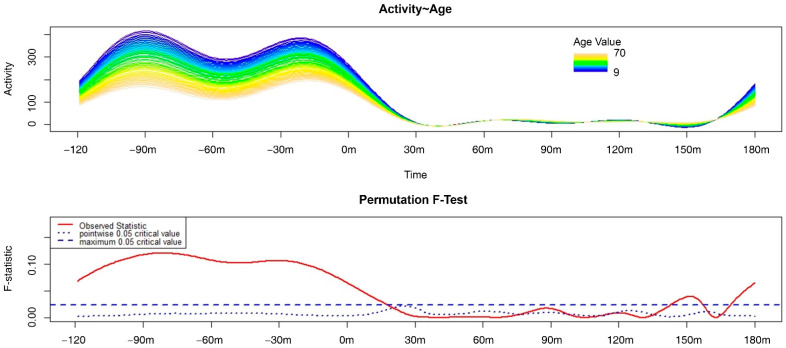
Results of the functional linear modeling applied to the exam of the variation in motor activity patterns of the wake–sleep transition according to age. In detail, the upper panel shows the variation in motor activity of each participant 120 min before and 180 min after bedtime according to age, with blue indicating the lowest and pink indicating the highest age. The lower panel shows the results of the non-parametric permutation F-test with significant differences when the solid red line (the observed statistics) is above both the dotted and dashed blue lines (the global and point-wise test of significance, respectively).

The variation in the motor activity pattern of the wake–sleep transition according to age (Figure 1) reached statistical significance at two times: one during the wake phase until 20 min after bedtime, the other around 150 min after bedtime. Regarding the first, a significantly higher level of motor activity was observed in younger participants compared to older participants within the whole wakefulness period considered (i.e., 120 min prior to bedtime) and during the 20 min after bedtime. With reference to the second interval, the older participants moved significantly more than the younger ones. This result confirms knowledge regarding the sleep architecture of the older population, which is characterized by greater vulnerability in terms of microawakenings and increased motor activity.

## 4. Discussion

The present study had two main aims: (1) to examine the time course of maWI dissipation; (2) to assess the potential role played by age in this dissipation. With reference to the first goal, we observed that maWI dissipated within 20 min, which was, overall, in line with our expectations. Regarding the second goal, we observed that age significantly modulated the maWI, with it being more evident in younger participants compared to older participants. Indeed, we observed a higher level of motor activity in younger individuals than in older individuals within the first 20 min after bedtime, which was in line with our expectations.

In an attempt to provide a meaningful explanation of the observed pattern of results, we made a comparison with data previously observed in the same sample on the dissipation of maSI [20] within the theoretical framework of the two-process model of sleep regulation [1,2]. Generally speaking, the pattern of results of the present study seems to suggest that maWI dissipation is comparable to that of maSI.

More specifically, while maSI dissipated in about one hour, maWI dissipated faster, in just 20 min. Therefore, it seems as if, at least from a motor point of view, it is easier to adjust to the wake–sleep transition than to the sleep–wake transition. This evidence disagrees with the well-known electrophysiological data describing sleep onset as a gradual process [30] and waking up as a binary process with electrophysiological changes occurring more quickly; actually, from a motor point of view, exactly the contrary seems to be the case. However, when trying to explain this discrepancy, we should keep in mind that we are considering two different types of signal, electrophysiological on one hand and motor on the other, thus, reducing their direct comparability. It is important to note that, in wake inertia, it is difficult to evaluate the start of the process in behavioral/cognitive terms. From this perspective, it would be interesting to study the pre-sleep period before going to bed with the lights off. The pre-sleep period could be important for understanding the link between general behavioral activity and maWI.

Taking the two-process model of sleep regulation as a reference, since the S process reaches its maximum value at sleep onset, we could suppose that it is highly involved in the faster dissipation of maWI. On the contrary, the slower dissipation of maSI could involve the C process more than the S process. Indeed, it is well known that this process oscillates within the 24 h period with a sinusoidal trend; more specifically, upon morning awakening, the values of the C process slowly rise, but they are far from the acrophase which is reached in the afternoon [1,2].

In order to test the plausibility of the greater involvement of the S and C processes in maWI and maSI dissipation, respectively, future studies should examine a sample balanced for age and social activities. More specifically, to further explore the role played by the C process, the variation in the motor activity at the sleep–wake and wake–sleep transitions at wake-up time and sleep-onset time, respectively, should be explored. Moreover, to quantify the contribution of the S process, it would be opportune to explore the variation in motor activity at the sleep–wake and wake–sleep transition compared to the total amount of sleep and wakefulness, respectively.

Some limitations of the current study should be disclosed, such as the different social habits of participants and the underrepresentation of older participants. However, some strengths should also be highlighted, such as the sample size being large, at least for a study based on the actigraphic technique, and this technique allowing the monitoring of maWI dissipation in a naturalistic setting, increasing the ecological validity of the work.

## 5. Conclusions

To the best of our knowledge, the present study has shown for the first time that maWI dissipates in 20 min, as well as demonstrated that the maWI phenomenon is much more evident in younger participants compared to older participants. This highlights the need for future studies specifically aimed at understanding the contribution of the two processes of sleep regulation in the dissipation of maWI and maSI. Finally, if this new way to assess, with the highest degree of ecological validity, both transitions (i.e., sleep/wake and wake/sleep) should be confirmed as reliable in future studies, new lines of research could be opened, presenting potential implications both in the basic (e.g., the models of sleep regulation) and clinical (e.g., sleep-onset disorder and/or sleep drunkenness) research fields.

## Data Availability

The data are not publicly available and cannot be shared due to ethical issues.
